# Androgen Receptors Act as a Tumor Suppressor Gene to Suppress Hepatocellular Carcinoma Cells Progression *via* miR-122-5p/RABL6 Signaling

**DOI:** 10.3389/fonc.2021.756779

**Published:** 2021-10-20

**Authors:** Neng Tang, Xiaolin Dou, Xing You, Yixiong Li, Xi Li, Guodong Liu

**Affiliations:** ^1^ Department of General Surgery, Xiangya Hospital, Central South University, Changsha, China; ^2^ Department of Geriatric Surgery, Xiangya Hospital, Central South University, Changsha, China; ^3^ National Clinical Research Center for Geriatric Disorders, Xiangya Hospital, Central South University, Changsha, China

**Keywords:** hepatocellular carcinoma, androgen receptor, invasion, migration, microRNA

## Abstract

Hepatocellular carcinoma (HCC) is a malignant tumor with a high degree of malignancy and a poor prognosis. Androgen receptor (AR) has been reported to play important roles in the regulation of the progression of HCC, but the underlying mechanisms of how AR regulates HCC initiation, progression, metastasis, and chemotherapy resistance still need further study. Our study found that AR could act as a tumor suppression gene to suppress HCC cells invasion and migration capacities *via* miR-122-5p/RABL6 signaling, and the mechanism study further confirmed that miR-122-5p could suppress the expression of RABL6 to influence HCC cells progression by directly targeting the 3’UTR of the mRNA of RABL6. The preclinical study using an *in vivo* mouse model with orthotopic xenografts of HCC cells confirmed the *in vitro* data, and the clinical data gotten from online databases based on TCGA samples also confirmed the linkage of AR/miR-122-5p/RABL6 signaling to the HCC progression. Together, these findings suggest that AR could suppress HCC invasion and migration capacities *via* miR-122-5p/RABL6 signaling, and targeting this newly explored signaling may help us find new therapeutic targets for better treatment of HCC.

## Introduction

Primary liver cancer is one of the main public health problems across the world, and HCC is its main pathological form. HCC is a malignant tumor with a high degree of malignancy and a poor prognosis, which ranks the fifth leading incidence of malignant tumors and the third leading cause of death in the world (1). In China, the main cause of HCC is hepatitis B virus infection (2). At present, the main treatment for early HCC is surgical resection, but unfortunately, most patients are already in the advanced stage at the time of diagnosis, especially in China (3). Therefore, further research on the detailed mechanisms of HCC invasion and metastasis and finding new therapeutic targets is crucial to improve the prognosis of HCC patients.

Sex hormones, especially androgen receptors (AR), have been reported to make a huge contribution for the development of HCC, but the results of the role of ARs in HCC seemed not consistent. Up to today, many researches believe that AR can promote tumorigenesis in the early stage of HCC, and inhibit its progression and metastasis in the late stage. Rogers, et al. reported that down regulated AR could reduce the incidence of carcinogen- and hepatitis B virus (HBV)-induced HCCs in mouse models remarkably (4). Recently, studies have shown that a variety of genetic molecules, such as microRNAs and circRNAs, are involved in the regulation of the HCC signaling pathway by AR (5–7). These findings demonstrated that AR played important roles in the regulation of the initiation, progression, and metastasis of HCC.

MicroRNAs (miRNAs) belong to the family of non-coding RNAs with 20-24 nucleotides in length. Generally, miRNAs can inhibit gene expression by binding to the 3’UTR of downstream genes through post-transcriptional regulation (6), and many studies have proven that miRNAs play an important role in the regulation of the initiation, progression and metastasis of many diseases (8–11).

RABL6 (RAB, member RAS oncogene family like 6), located at 9q34.3 (12), which encodes a member of the Ras superfamily of small GTPases. The encoded protein binds to both GTP and GDP and may play a role in cell growth and survival. Recently, studies report that RABL6 was highly expressed in many cancers, such as non-small cell lung cancer (NSCLC), breast cancer, and pancreatic ductal adenocarcinoma (PDAC), and often related to the poor prognosis for the patients (12–15), which indicated that RABL6 may act as an oncogene to promote cancer progression.

Here, our study showed that AR could suppress liver cancer cells invasion and proliferation capacities *via* miR-122-5p/RABL6 signaling, and miR-122-5p could suppress the expression of RABL6 to influence liver cancer cells progression by directly targeting the 3’UTR of RABL6-mRNA. Thus, we find a new pathway between AR and liver cancer progression, and may provide new strategies to better suppress liver cancer progression.

## Materials and Methods

### Cell Culture

Human HCC Hep3B and PLC/PRF/5 cells and HEK 293T cells were cultured in Dulbecco’s Modified Eagle’s Medium (DMEM, Invitrogen) with 10% Fetal Bovine Serum (FBS), 1% penicillin/streptomycin, and 1% glutamine. All cells were cultured in a 37°C temperate box in a 5% (v/v) CO2 condition.

### Invasion Assay

1x10^5^ HCC cells were seeded into an 8 μm transwell chamber (Corning Life Science) with 100 μl diluted Matrigel (BD Corning) coated first in a serum free medium, then the bottom chamber was filled with 700 μL medium (10% FBS), and after being cultured for 24 hours, we collected the transwell chamber for fixation and dyeing of the invaded cells, then observed and counted the cells under the microscope. Each sample was designed in three duplicate chambers and several pictures under different horizons were taken for analysis.

### Wound Healing Migration Assay

The HCC cells were seeded in a 6 well plate, and when the cells were more than 90% full, a sterile tip was used to make a scratch and it was set as 0h. Pictures were taken with a microscope and record. After being incubated for 24 hours in the incubator, pictures were taken again and the distance of cell migration was measured. Each sample was designed in three duplicate wells and several pictures under different horizons were taken for analysis.

### MTT Assay

Firstly, the culture medium containing 10% fetal calf serum was used to prepare a single cell suspension and inoculate 5000 cells per well, with a volume of 200ul per well in a 96-well plate. Secondly, the cells were cultured for 1-6 days respectively in the general cell culture conditions. Thirdly, 20ul of MTT solution (5mg/ml in PBS, pH=7.4) was added to each well and continued incubating for 4 hours. Fourthly, the culture supernatant was carefully aspirated and discarded in the well and 150ul DMSO was added to each hole and shaken for 10 minutes to fully melt the crystals. Fifthly, the light absorption value of each hole was measured on the enzyme-linked immunosorbent monitor at 490nm, the result recorded, and the cell growth curve drawn with the time as the abscissa and the absorbance as the ordinate.

### Colony Formation Assay

Firstly, the cells were digested in the logarithmic growth phase with 0.25% trypsin and a pipette into single cells, and suspended in DMEM culture medium of 10% fetal bovine serum for later use. Secondly, 300 cells were added to a six-well with 3ml of culture medium, gently rotated to mix the cells, and then incubated in a cell culture incubator for 2 weeks. Thirdly, the supernatant was discarded, and carefully washed twice with PBS. 4% paraformaldehyde was added to fix for 15 minutes, the fixative removed, and GISMA was added for dyeing for 10-30 minutes, then the dyeing solution was slowly washed off with running water and dried in the air. Fourthly, the clone formation rate was counted and calculated. Clone formation rate = (number of clones/number of inoculated cells)x100%.

### Western Blot Assay

30 ug proteins were loaded into 10% SDS/PAGE gel and then transferred onto a PVDF membrane. After being incubated with the primary antibody in a 4°C condition for overnight and secondary antibody at room temperature for 1-2 hours, the membrane was visualized with an ECL system. The antibodies information are listed below: RABL6 (PA5-31048, Thermo Fisher Scientific), CPEB1 (ab127739, Abcam), RPL4 (PA5-98127, Thermo Fisher Scientific), CLIC5 (ab191102, Abcam), NOL4L (ab237758, Abcam), GAPDH (ab9485, Abcam).

### qRT-PCR Assay

Total RNA was extracted from tissues and cells using TRIzol reagent (Invitrogen) and subjected to reverse transcription with a TakaRa PrimeScript™ RT kit (Takara, Dalian, China). qRT-PCR assay was performed with the SYBR^®^ Premix Ex Taq™ II (Takara, Dalian, China) in a CFX96 Touch™ real-time PCR detection system (Bio-Rad Laboratories, Hercules, CA, USA) following the instructions. The expression of miRNAs was measured by TaqMan miRNA assays (Applied Biosystems, Foster City, CA, USA). All the primers were ordered from IDT Company.

### Cell Transfection

The pLKO.1-shAR, pWPI-oeAR, pLV-miR-122-5p (oemiR-122-5p), pLKO.1-shRABL6, pWPI-RABL6 plasmids, the psPAX2 packaging plasmid, and pMD2.G envelope plasmid were transfected into HEK-293T cells using the standard calcium chloride transfection method for 48 h to get the lentivirus soup. The lentivirus soups were collected and concentrated by density gradient centrifugation and used immediately or frozen at -80°C for later use. Lentivirus soups were added into the medium until the cells were 60% confluent, polybrene (final concentration was 10 ug/ml) was added to improve transfection efficiency. 24 hours later, the medium was refreshed and transfected with lentivirus soups again. The transfected cells were used for further experiments after the transfection efficiency had been checked.

### Luciferase Assay

683bp of RABL6 3’UTR was chosen and the potential binding site of miR-122-5p was obtained by using the miRDB and Genome databases. Based on which, we constructed the wild type and mutant (the binding site was replaced by GGATCCG) RABL6 3’UTR psiCHECK-2 plasmids. Then, the HCC cells were transduced with the wild type/mutant plasmids and pLV/oemiR-122-5p or control/miR-122-5p inhibitor (Thermo Fisher Scientific). After being cultured for 48 hours, the cells were collected and the luciferase activity was measured by a dual luciferase reporter assay according to the protocol.

### *In Vivo* Studies

Six to eight week old nude mice were purchased from NCI and divided into three groups for injection of 1 × 10^6^ PLC/PRF/5 cells transduced with Luciferase (luc) and with/without shAR and oemiR-122-5p for 3 groups (1: pLKO+pLV; 2: shAR+pLV; 3: shAR+oemiR-122-5p). The various PLC/PRF/5 cells at 1 × 10^6^ were mixed with Matrigel (1:1) and injected into the mice under the left lobes of the liver capsules. Tumor development and metastasis were monitored by non-invasive *In Vivo* Fluorescent Imager (IVIS Spectrum, Caliper Life Sciences) once a week. Mice were sacrificed after 8 weeks, and tumors and any metastases were removed for studies. The study was carried out under the approval of the ethics committee of Xiangya Hospital Central South University and followed the Interdisciplinary Principles and Guidelines for the Use of Animals in Research, Testing, and Education by the New York Academy of Sciences, *Ad Hoc* Animal Research Committee.

### Statistics

All statistical analysis was conducted by SPSS 22.0 software, and the data were shown as mean ± SD. Differences in mean values between two groups were analyzed by two tailed Student’s t test and the mean values of more than two groups were compared with one-way ANOVA. We thought it as significant difference when p ≤ 0.05.

## Results

### AR Could Suppress HCC Cells Invasion and Migration Capacities

**Figure 1:**
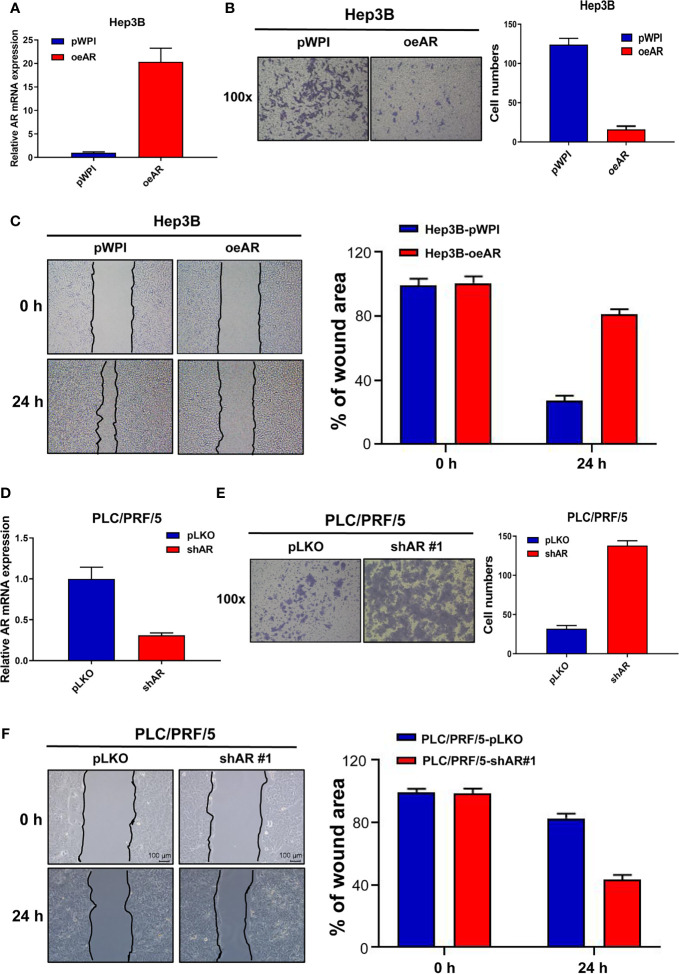
AR could suppress HCC cells invasion and migration capacities. **(A)** qRT-PCR assay was used to check AR mRNA expression after overexpressing AR in HCC Hep3B cells. **(B)** Transwell invasion assay was used to check HCC Hep3B cells invasion capacity after oeAR in the cells. **(C)** Wound healing migration assay was used to check HCC Hep3B cells migration capacity after oeAR in the cells. **(D)** qRT-PCR assay was used to check AR mRNA expression after knocking down AR in HCC PLC/PRF/5 cells. **(E)** Transwell invasion assay was used to check HCC PLC/PRF/5 cells invasion capacity after shAR in the cells. **(F)** Wound healing migration assay was used to check HCC PLC/PRF/5 cells migration capacity after shAR in the cells.

To check AR’s role in the regulation of HCC progression, we first manipulated AR (over-express or knock down) in HCC Hep3B and PLC/PFR/5 cells, then we conducted transwell invasion assay and wound healing migration assay to check the cells’ invasion and migration capacities. The results showed that oeAR (over-expressing AR) could decrease HCC Hep3B cells’ invasion and migration capacities (**Figures 1A–C**), and shAR (knocking down AR) led to the increase of the invasion and migration capacities of HCC PLC/PRF/5 cells (**Figures 1D–F**), which was also consistent with our previous data (16, 17). At the same time, we constructed the 2# shAR plasmid to knock down AR in HCC PLC/PRF/5 cells, and the results showed which could also increase the cells invasion and migration capacities, but the promotion effect was not as strong as the 1# shAR plasmid(**Supplementary Figures 1A, B**), so we chose the 1# shAR plasmid for further study. To study the function of ARs in the same HCC cells, we also studied shAR in HCC Hep3B cells and oeAR in HCC PLC/PRF/5 cells, and the results of the invasion and migration capacities were also consistent with our previous data in **Figure 1** and **Supplementary Figures 1C–E**.

Together, the data from **Figure 1** and **Supplementary Figure 1** indicated that AR could suppress HCC cells invasion and migration capacities.

### AR May Decrease HCC Progression *via* Altering miR-122-5p Expression

**Figure 2:**
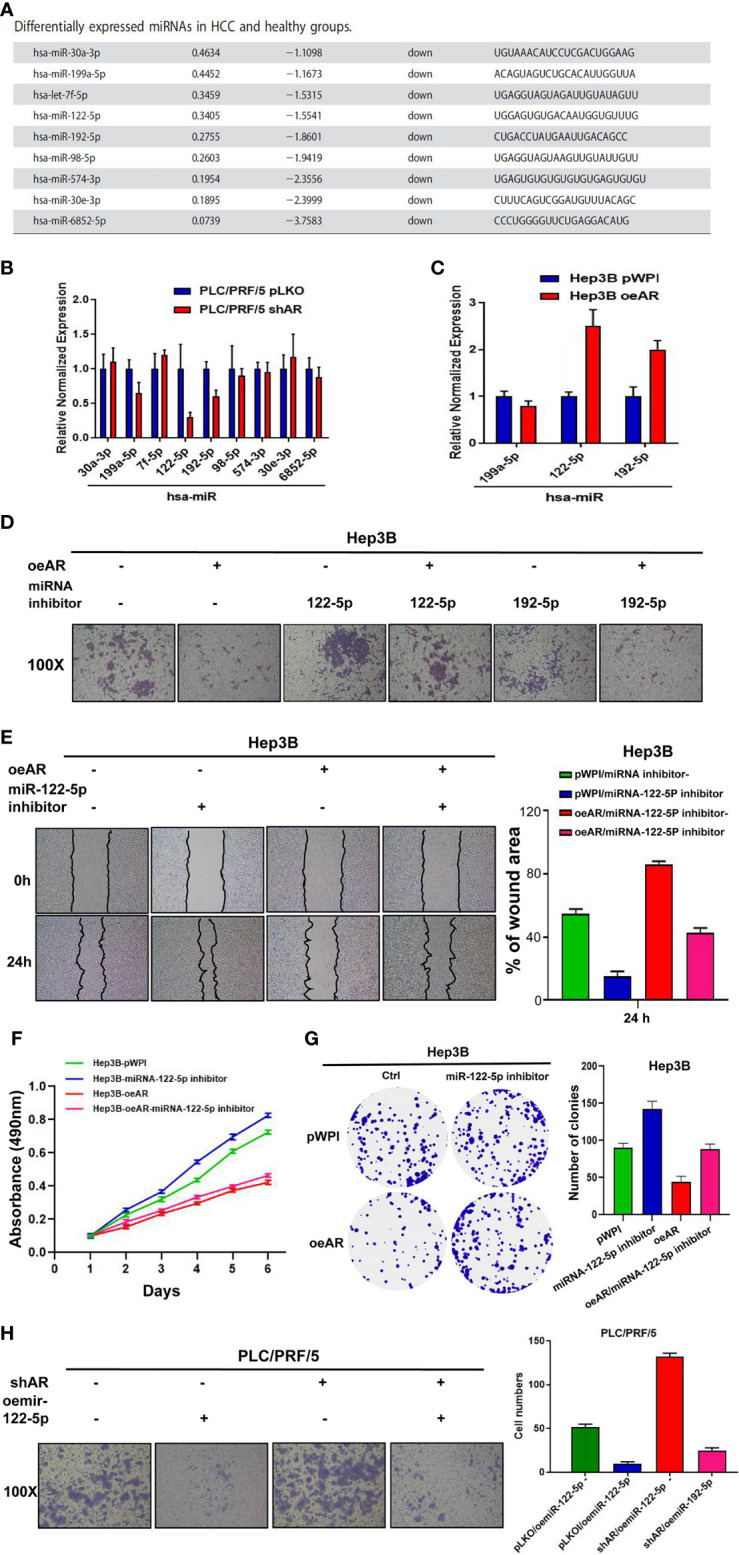
AR may decrease HCC progression *via* altering miR-122-5p expression. **(A)** 9 downregulated miRNAs was selected from the published papers. **(B, C)** qRT-PCR assay was used to check the miRNAs expression after shAR/oeAR in HCC cells. **(D)** Transwell invasion assay was used to check the invasion capacity of HCC Hep3B cells transfected with pWPI/oeAR after adding miR-122-5p inhibitor or miR-192-5p inhibitor in the cells. **(E)** Wound healing migration assay was used to check HCC Hep3B cells migration capacity after oeAR/adding miR-122-5p inhibitor in the cells. **(F, G)** MTT and colony formation assays were used to check HCC Hep3B cells proliferation capacities after oeAR/adding miR-122-5p inhibitor in the cells. **(H)** Transwell invasion assay was used to check HCC PLC/PRF/5 cells invasion capacity after shAR/oemiR-122-5p in the cells.

To study the detailed mechanism between AR and HCC, and find a new pathway to provide new strategies to better suppress HCC progression, we focused on miRNAs, for the reason that many papers have reported that miRNAs might play important roles in the regulation of progression of many cancers (8–11). We first searched the published papers related to RNA sequencing in HCC, and selected 9 miRNAs that were reported to be downregulated in HCC compared to healthy group (18) (**Figure 2A**). Then we checked the miRNAs expression after knocking down/overexpression AR in HCC cells, and the results showed that 3 of 9 miRNAs (hsa-miR-122-5p, miR-192-5p, miR-199a-5p) were decreased after shAR in HCC PLC/PRF/5 cells, and 2 of the 3 miRNAs (hsa-miR-122-5p and miR-192-5p) were increased after oeAR in HCC Hep3B cells (**Figures 2B, C**). Next, we checked the miR-122-5p and miR-192-5p functions to HCC cells after adding miRNAs inhibitor or overexpressing miRNAs. The results showed that adding miR-122-5p inhibitor could partly reverse AR’s role in the regulation of the invasion capacity of HCC Hep3B cells, but not miR-192-5p inhibitor (**Figure 2D**), and wound healing migration assay also confirmed miR-122-5p’s role in HCC progression (**Figure 2E**). At the same time, we checked the proliferation capacities of the HCC cells by using MTT assay and colony formation assay, and the results also showed that oeAR could decrease the proliferation capacities of HCC Hep3B cells, and adding miR-122-5p inhibitor could partly reverse AR’s role in the regulation of the proliferation capacity of HCC Hep3B cells (**Figures 2F, G**). We also examined oemiR-122-5p in HCC PLC/PRF/5 cells to check its function, and the data showed that adding miR-122-5p could block shAR’s role in promoting HCC cells invasion, migration, and proliferation capacities (**Figure 2H** and **Supplementary Figures 2A–C**).

Together, the data from **Figure 2** and **Supplementary Figure 2** indicated that AR may decrease HCC invasion, migration, and proliferation capacities *via* altering miR-122-5p expression.

### AR/miR-122-5p Axis May Influence HCC Cells Invasion, Migration, and Proliferation Capacities *via* Altering the RABL6 Expression

**Figure 3:**
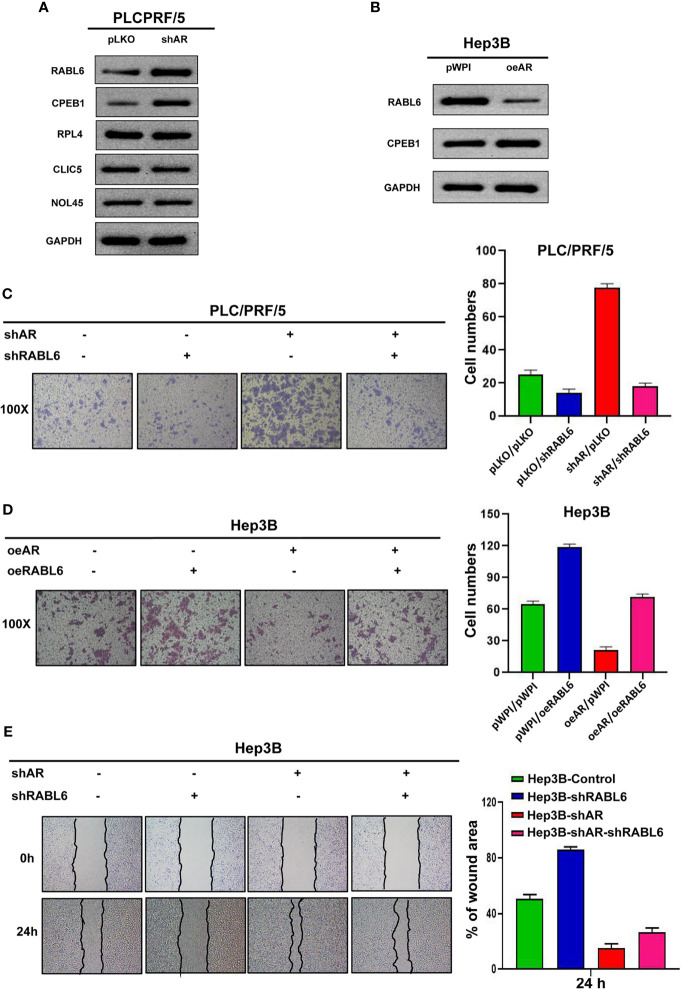
AR/miR-122-5p axis may influence HCC cells invasion and migration capacities *via* altering the RABL6 expression. **(A, B)** Western blot assay was used to check related gene protein expression after shAR/oeAR in HCC cells. **(C)** Transwell invasion assay was used to check HCC PLC/PRF/5 cells invasion capacity after shAR/shRABL6 in the cells. **(D)** Transwell invasion assay was used to check HCC Hep3B cells invasion capacity after oeAR/oeRABL6 in the cells. **(E)** Wound healing migration assay was used to check HCC Hep3B cells migration capacity after oeAR/oeRABL6 in the cells.

To check the underlying mechanism of how AR/miR-122-5p signaling regulates HCC cells progression, we searched the miRDB database (http://mirdb.org/index.html) and predicted 5 potential genes (RABL6, CPEB1, RPL4, CLIC5, NOL4L) that targeted by miR-122-5p. Then we checked these genes’ expression after shAR/oeAR in HCC Hep3B or PLC/PRF/5 cells. The results from **Figures 3A, B** showed that RABL6 may be the potential gene that is regulated by AR. Next we conducted the reverse assay to check if shRABL6 could reverse shAR’s function on HCC cells progression. The result showed that shRABL6 could reverse shAR’s function on HCC PLC/PRF/5 cells invasion and proliferation capacities (**Figure 3C** and **Supplementary Figures 3A, B**), and at the same time, oeRABL6 could reverse oeAR’s function on HCC Hep3B cells invasion, migration, and proliferation capacities (**Figures 3D, E** and **Supplementary Figures 3C, D**).

Together, the data from **Figure 3** and **Supplementary Figures 3A–D** indicated that AR/miR-122-5p axis may influence HCC cells invasion and migration capacities *via* altering the RABL6 expression.

### MiR-122-5p Directly Targets 3’UTR of RABL6 mRNA to Regulate RABL6 Expression

**Figure 4:**
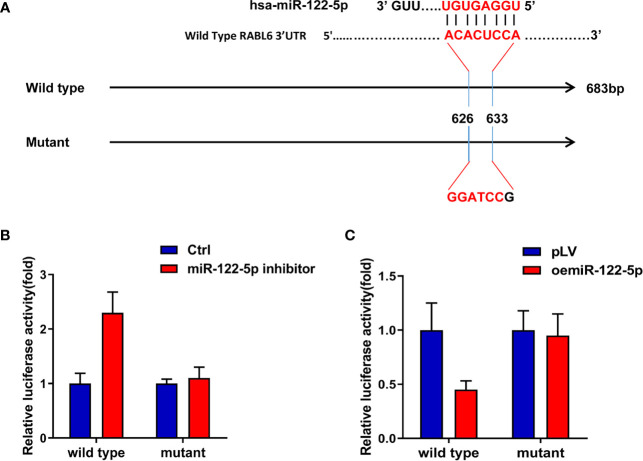
MiR-122-5p directly targets 3’UTR of RABL6 mRNA to regulate RABL6 expression. **(A)** Pattern diagram of the design of mutant type 3’UTR of RABL6. **(B)** Luciferase activities were measured when adding miR-122-5p inhibitor in Hep3B wild type and mutant cells compared to the control cells. **(C)** Luciferase activitIes were measured when overexpressing miR-122-5p in PLC/PRF/5 RABL6 wild type and mutant cells compared to the control cells.

As the Argonaute 2 IP assay indicated miRNAs participated in the AR and RABL6 pathway (**Supplementary Figures 3E, F**), and our data proved miR-122-5p was the candidate miRNA, so, to check how miR-122-5p regulate RABL6 expression, we first searched the miRDB database (http://mirdb.org/index.html) to find out the binding sites of miR-122-5p on the 3’UTR of RABL6 mRNA and constructed wild type and mutant sequences (**Figure 4A**), and the results from the luciferase assay indicated that the luciferase activity was increased when adding the miR-122-5p inhibitor in HCC Hep3B RABL6 wild type cells compared to the control cells, but the activity in the mutant groups did not increase significantly when compared to the control group (**Figure 4B**).What is more, the luciferase activity decreased significantly when oemiR-39-5p was found in HCC PLC/PRF/5 cells in the wild type group but not mutant groups (**Figure 4C**).

Together, the data of **Figures 4A–C** proved that miR-122-5p could directly target 3’UTR of RABL6 mRNA to regulate the expression of RABL6.

### Clinical Data to Confirm the Clinical Significance of AR/miR-122-5p/RABL6 Axis in the Progress of HCC

**Figure 5:**
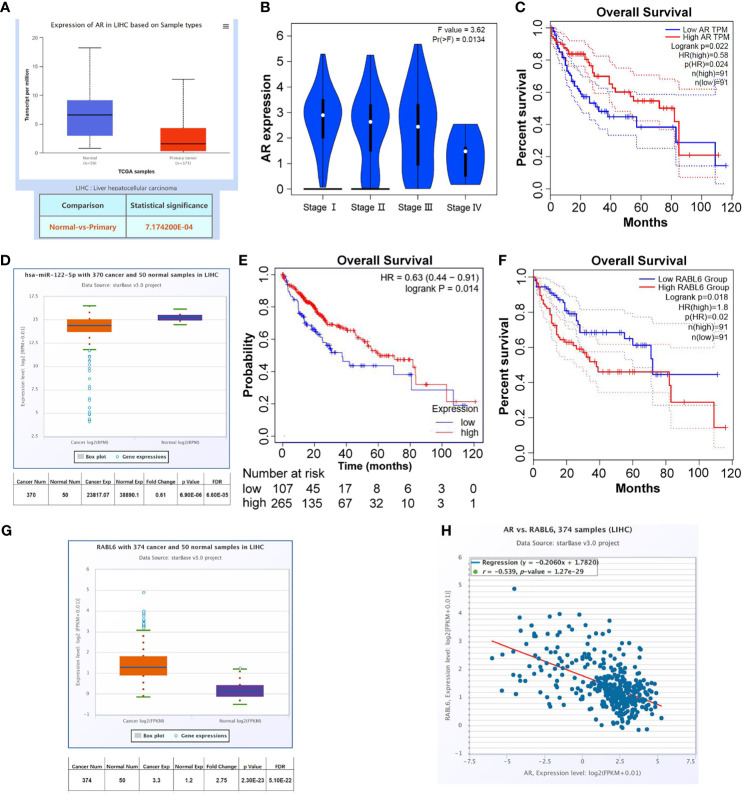
Clinical data to confirm the clinical significance of AR/miR-122-5p/RABL6 axis in the progress of HCC. **(A)** UALCAN database was used to check AR expression in normal tissues and LIHC samples. **(B)** GEPIA database was used to check AR expression in different stages of LIHC patients. **(C)** GEPIA database was used to analyze survival of different expression of AR in LIHC patients. **(D)** ENCORI database was used to check miR-122-5p expression in normal tissues and LIHC samples. **(E)** Kaplan-Meier Plotter database was used to analyze survival of different expression of miR-122-5p in LIHC patients. **(F)** GEPIA database was used to analyze survival of different expression of RABL6 in LIHC patients. **(G)** ENCORI database was used to check RABL6 expression in normal tissues and LIHC samples. **(H)** ENCORI database was used to analyze the expression correlation between AR and RABL6.

To check the clinical significance of AR/miR-122-5p/RABL6 axis in liver cancer, we collected the data from UALCAN (http://ualcan.path.uab.edu/index.html), Kaplan-Meier Plotter (http://www.kmplot.com/analysis/index.php?p=service), GEPIA (http://gepia.cancer-pku.cn/index.html), and ENCORI (http://starbase.sysu.edu.cn/panCancer.php) databases. The results showed that AR expression was decreased in primary tumors (**Figure 5A**), and as the stages increase, the expression of AR gradually decreases (**Figure 5B**). What is more, the data from GEPIA database (Quartile) showed that low expression of AR in LIHC patients often meant worse survival rate (**Figure 5C**). At the same time, the data from ENCORI and Kaplan-Meier Plotter databases showed that miR-122-5p expression was decreased in LIHC patients and low expression of miR-122-5p often meant worse survival rate for LICH patients (**Figures 5D, E**). What’s more, the data from ENCORI and GEPIA databases concluded that RABL6 expression was increased in LIHC patients and high expression of RABL6 was connected to low survival rate in LIHC patients (**Figures 5F, G**). The co-expression analysis through ENCORI database proved that AR and RABL6 expression were negatively correlated (**Figure 5H**), which was consist with our *in vitro* data.

Together, the data from **Figures 5A–H** indicated that AR/miR-122-5p/RABL6 axis played important roles in the progression of liver cancer.

### Preclinical Study Using *In Vivo* Mouse Model to Test the Role of AR/miR-122-5p/RABL6 in HCC Cell Progression

**Figure 6:**
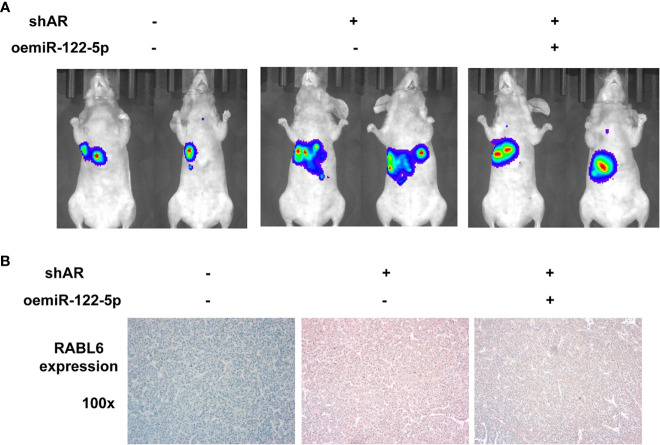
Preclinical study using *in vivo* mouse model to test the role of AR/miR-122-5p/RABL6 in HCC cell progression. **(A)** IVIS imaging was used to detect the various distal metastasis foci in mice with orthotopically xenografted tumor cells under the left lobes of the liver capsule. Two representative mouse IVIS bioluminescent images are shown for each group. **(B)** Representative images of IHC staining for RABL6 in the three groups of mice at ×100 magnification.

To examine the validity of the above *in vitro* cell line data in the *in vivo* mouse model, we applied the orthotopic HCC xenograft mouse model. We generated HCC PLC/PRF/5 cells with luciferase (luc) expression with/without shAR and oemiR-122-5p for 3 groups (1: pLKO+pLV; 2: shAR+pLV; 3: shAR+oemiR-122-5p). The HCC cells were inoculated into the left lobes of liver capsule of nude mice and tumor size and metastases were monitored weekly *via* the non-invasive *In Vivo* Imaging Systems (IVIS) for 8 weeks. The results revealed that shAR could promote the tumor progression in the mouse model, and oemiR-122-5p could partly reverse AR’s function in the mouse tumor model **(** **Figure 6A** **)**. Importantly, results from IHC staining demonstrated that shAR led to increase the RABL6 expression, which could be partly reversed after oemiR-122-5p (**Figure 6B**).

Together, results from our preclinical study using an *in vivo* mouse model in **Figure 6** prove that AR may play a protective role to suppress the HCC progression *via* altering the AR/miR-122-5P/RABL6 axis.

## Discussion

Liver cancer is a malignant tumor with the fifth highest incidence rate and second highest mortality rate in the United States, and its incidence is increasing year by year (19), and the incidence of liver cancer is higher in the developing countries (20). There are many risk factors for liver cancer, including hepatitis B virus, hepatitis C virus, fatty liver disease, alcohol-related cirrhosis, smoking, obesity, diabetes, iron overload, and various dietary exposures (21). The prognosis of liver cancer is very poor, usually only 10-15% of patients can be treated with surgery, the reason for which being that most patients are in the advanced stage of the disease at the time of diagnosis. Treatment options for advanced liver cancer include trans-arterial chemoembolization (TACE), chemotherapy, and immunotherapy, etc. Sorafenib has been proven to have a certain effect on some patients with advanced liver cancer, but its drug resistance is a problem that doctors have to face (22). So, further study of the molecular signaling pathways of liver cancer invasion and metastasis, and the discovery of new therapeutic targets to improve the prognosis of liver cancer patients are necessary.

Sex hormones and their receptors, especially androgen receptors, have been confirmed to play an important role in the occurrence, development, and metastasis of HCC, but the specific mechanisms still need to be further studied (23, 24). Studies have shown that AR degradation enhancer, ASC-J9, may have a certain effect on the treatment of liver cancer, but it still remains controversial (25, 26). Research has shown that ARs have dual roles in HCC. They found that ARs might enhance HCC initiation and early development, but suppress HCC metastasis at the later stages of the disease (27–29). Our research is another study to show a new mechanism signaling between ARs and HCC, and also prove that ARs can work as a tumor suppressor to regulate HCC progression.

MicroRNAs (miRNAs) are short (20-24 nt) non-coding RNAs that are involved in post-transcriptional regulation of gene expression in multicellular organisms by affecting both the stability and translation of mRNAs. miRNAs have been proven to play important roles in the regulation of the progression and metastasis in many diseases. In our study, we selected the related miRNAs based on published sequencing data (18), and confirmed miR-122-5p played important role in the AR and RABL6 pathway in HCC progression. Many studies have reported miR-122-5p participated in the regulation of many cancers’ progression and played important roles in the prognosis in these cancers. Wang et al. reported that overexpressed miR-122-5p promotes cell viability, proliferation, migration, and glycolysis of renal cancer by negatively regulating PKM2 (30). Ding et al. showed that miR-122-5p modulates the radiosensitivity of cervical cancer cells by regulating cell division cycle 25A (CDC25A) (31). Our study was the first time to study miR-122-5p’s role in the HCC progression under the regulation of AR, which indicated that miR-122-5p may also function in the sex hormones signaling in HCC initiation, progression and metastasis. MiRNAs may influence the expression of targeted genes through different regulations, and among which, the post-transcriptional regulation has been proven to be the most common form of regulation. In our study, through sequence analysis, target prediction, and dual luciferase report assay, we also concluded that miR-122-5p could regulate downstream gene RABL6 expression by directly target the 3’UTR of RABL6-mRNA, which also confirmed that miR-122-5p could regulate target genes expression through the common regulation manner.

RABL6 is a novel Ras superfamily protein. The Ras superfamily of GTPases comprises several subfamilies of small GTP-binding proteins which play pivotal roles in tumorigenesis, as their functions included cell proliferating, differentiating, and apoptosis (32, 33). Several studies have reported that RABL6 was highly expressed in many cancers, such as non-small cell lung cancer (NSCLC), breast cancer, and pancreatic ductal adenocarcinoma (PDAC), and is often related to the poor prognosis for the patients (10–12). Our study was the first to check RABL6’s role in HCC, and found that RABL6 also could work as an oncogene to promote HCC progression, which was consistent with its role in other cancers. What is more, the reverse assay also confirmed that RABL6 could influence HCC progression under the regulation of AR. At the same time, RABL6 is known to endorse protein binds to both GTP and GDP and may play a role in cell growth and survival. In our study, we also verified the proliferation capacity through MTT assay and colony formation assay, and confirmed that RABL6 could not only promote the invasion and migration abilities of HCC cells, but also promote their proliferation ability, under the regulation of AR/miR-122-5p signaling.

In conclusion, our study found that AR could suppress liver cancer cells invasion and proliferation capacities *via* miR-122-5p/RABL6 signaling, and miR-122-5p could suppress the expression of RABL6 to influence liver cancer cells progression by directly targeting the 3’UTR of RABL6-mRNA. Thus, we find a new pathway between AR and liver cancer progression, and targeting this new pathway may provide us with better therapies for HCC.

## Data Availability Statement

The original contributions presented in the study are included in the article/**Supplementary Material**. Further inquiries can be directed to the corresponding authors.

## Author Contributions

Conceptualization, GL, XL and NT. Experiments, GL, NT, XL, XD, XY and YL. Data analysis, NT, XL, XY and YL. Writing, GL, NT, XL, XY and YL. Supervision, GL, NT, XL, XL, XY and YL. Funding Acquisition, GL. All authors contributed to the article and approved the submitted version.

## Funding

This work was supported by The Youth Science Foundation of Xiangya Hospital (2020Q09).

## Conflict of Interest

The authors declare that the research was conducted in the absence of any commercial or financial relationships that could be construed as a potential conflict of interest.

## Publisher’s Note

All claims expressed in this article are solely those of the authors and do not necessarily represent those of their affiliated organizations, or those of the publisher, the editors and the reviewers. Any product that may be evaluated in this article, or claim that may be made by its manufacturer, is not guaranteed or endorsed by the publisher.

## Supplementary Material

Supplementary Figure 1
**(A)** Transwell invasion assay was used to check the cells invasion capacity after transfecting the second shAR plasmid into the HCC PLC/PRF/5 cells. **(B)** Would healing migration assay was used to check the cells migration capacity after transfecting the second shAR plasmid into the HCC PLC/PRF/5 cells. **(C)** Transwell invasion assay was used to check the cells invasion capacity after knocking down AR in HCC Hep3B cells. **(D)** Would healing migration assay was used to check the cells migration capacity after knocking down AR in HCC Hep3B cells. **(E)** Transwell invasion assay was used to check the cells invasion capacity after overexpressing AR HCC PLC/PRF/5 cells.

Supplementary Figure 2
**(A)** Wound healing migration assay was used to check HCC PLC/PRF/5 cells migration capacity after shAR/oemiR-122-5p in the cells. **(B, C)** MTT and colony formation assays were used to check HCC PLC/PRF/5 cells proliferation capacity after shAR/oemiR-122-5p in the cells.

Supplementary Figure 3
**(A, B)** MTT and wound healing assays were used to check HCC PLC/PRF/5 cells proliferation capacity after shAR/shRABL6 in the cells. **(C, D)** MTT and wound healing assays were used to check HCC Hep3B cells invasion capacity after oeAR/oeRABL6 in the cells. **(E, F)** The RABL6 mRNA level was detected in Argonaute 2 complex using RNA interaction-precipitation (RIP) assay in Hep3B **(E)** cells transfected with pWPI or oeAR and in PLC/PRF/5 **(F)** cells transfected with pLKO or shAR.

## References

[1] Forner A Reig M Bruix J. Hepatocellular Carcinoma. Lancet (2018) 391:1301–14. doi: 10.1016/S0140-6736(18)30010-2 29307467

[2] Chen W Zheng R Baade PD Zhang S Zeng H Bray F. Cancer Statistics in China, 2015. CA Cancer J Clin (2016) 66(2):115–32. doi: 10.3322/caac.21338 26808342

[3] Raza A Sood GK. Hepatocellular Carcinoma Review: Current Treatment, and Evidence-Based Medicine. World J Gastroenterol (2014) 20(15):4115–27. doi: 10.3748/wjg.v20.i15.4115 PMC3989948 24764650

[4] Rogers AB Theve EJ Feng Y Fry RC Taghizadeh K Clapp KM. Hepatocellular Carcinoma Associated With Livergender Disruption in Male Mice. Cancer Res (2007) 67(24):11536–46. doi: 10.1158/0008-5472.CAN-07-1479 18089782

[5] Han ZG. Functional Genomic Studies: Insights Into the Pathogenesis of Liver Cancer. Annu Rev Genomics Hum Genet (2012) 13:171–205. doi: 10.1146/annurev-genom-090711-163752 22703171

[6] Yu Z Cheng AS. Epigenetic Deregulation of microRNAs: New Opportunities to Target Oncogenic Signaling Pathways in Hepatocellular Carcinoma. Curr Pharm Des (2013) 19(7):1192–200. doi: 10.2174/138161213804805757 23092339

[7] Liu M Jiang L Guan XY. The Genetic and Epigenetic Alterations in Human Hepatocellular Carcinoma: A Recent Update. Protein Cell (2014) 5(9):673–91. doi: 10.1007/s13238-014-0065-9 PMC4145080 24916440

[8] Ma T Hu Y Guo Y Yan B. Tumor-Promoting Activity of Long Non-Coding RNA LINC00466 in Lung Adenocarcinoma *via* microRNA-144–Regulated HOXA10 Axis. Am J Pathol (2019) 189(11):2154–70. doi: 10.1016/j.ajpath.2019.06.014 31381886

[9] Wang W Zhou R Wu Y Liu Y Su W Xiong W. PVT1 Promotes Cancer Progression *via* MicroRNAs. Front Oncol (2019) 9:609. doi: 10.3389/fonc.2019.00609 31380270 PMC6644598

[10] Sárközy M Gáspár R Zvara Á Kiscsatári L Varga Z Kővári B. Selective Heart Irradiation Induces Cardiac Overexpression of the Pro-Hypertrophic miR-212. Front Oncol (2019) 9:598. doi: 10.3389/fonc.2019.00598 31380269 PMC6646706

[11] Liu J Wang P Zhang P Zhang X Du H Liu Q. An Integrative Bioinformatics Analysis Identified miR-375 as a Candidate Key Regulator of Malignant Breast Cancer. J Appl Genet (2019) 60(3-4):335–46. doi: 10.1007/s13353-019-00507-w 31372832

[12] Muniz VP Askeland RW Zhang X Reed SM Tompkins VS Hagen J. RABL6A Promotes Oxaliplatin Resistance in Tumor Cells and Is a New Marker of Survival for Resected Pancreatic Ductal Adenocarcinoma Patients. Genes Cancer (2013) 4(7–8):273–84. doi: 10.1177/1947601913501074 PMC3807645 24167655

[13] Cheng KW Lahad JP Kuo WL Lapuk A Yamada K Auersperg N. The RAB25 Small GTPase Determines Aggressiveness of Ovarian and Breast Cancers. Nat Med (2004) 10(11):1251–6. doi: 10.1038/nm1125 15502842

[14] Li YY Fu S Wang XP Wang HY Zeng MS Shao JY. Down-Regulation of C9orf86 in Human Breast Cancer Cells Inhibits Cell Proliferation, Invasion and Tumor Growth and Correlates With Survival of Breast Cancer Patients. PLoS One (2013) 8(8):e71764. doi: 10.1371/journal.pone.0071764 23977139 PMC3743754

[15] Yoshimura K Osman M Inoue Y Suda T Sugimura H. A Novel Prognostic Marker of Non-Small Cell Lung Cancer: Chromosome 9 Open Reading Frame 86 (C9orf86). J Thorac Dis (2016) 8(9):2284–6. doi: 10.21037/jtd.2016.08.38 PMC5059359 27746955

[16] Xiao Y Sun Y Liu G Zhao J Gao Y Yeh S. Androgen Receptor (AR)/miR-520f-3p/SOX9 Signaling Is Involved in Altering Hepatocellular Carcinoma (HCC) Cell Sensitivity to the Sorafenib Therapy Under Hypoxia *via* Increasing Cancer Stem Cells Phenotype. Cancer Lett (2019) 444:175–87. doi: 10.1016/j.canlet.2018.11.004 30448543

[17] Liu G Ouyang X Sun Y Xiao Y You B Gao Y. The miR-92a-2-5p in Exosomes From Macrophages Increases Liver Cancer Cells Invasion *via* Altering the AR/PHLPP/p-AKT/β-Catenin Signaling. Cell Death Differ (2020) 27(12):3258–72. doi: 10.1038/s41418-020-0575-3 PMC7853149 32587378

[18] Tan Y Ge G Pan T Wen D Chen L Yu X. A Serum microRNA Panel as Potential Biomarkers for Hepatocellular Carcinoma Related With Hepatitis B Virus. PLoS One (2014) 9(9):e107986. doi: 10.1371/journal.pone.0107986 25238238 PMC4169601

[19] Siegel RL Miller KD Jemal A. Cancer Statistics, 2019. CA Cancer J Clin (2019) 69(1):7–34. doi: 10.3322/caac.21551 30620402

[20] Starley BQ Calcagno CJ Harrison SA. Nonalcoholic Fatty Liver Disease and Hepatocellular Carcinoma: A Weighty Connection. Hepatology (2010) 51(5):1820–32. doi: 10.1002/hep.23594 20432259

[21] Center MM Jemal A. International Trends in Liver Cancer Incidence Rates. Cancer Epidemiol Biomarkers Prev (2011) 20(11):2362–8. doi: 10.1158/1055-9965.EPI-11-0643 21921256

[22] El-Serag HB Marrero JA Rudolph L Reddy KR. Diagnosis and Treatment of Hepatocellular Carcinoma. Gastroenterology (2008) 134(6):1752–63. doi: 10.1053/j.gastro.2008.02.090 18471552

[23] Shi L Feng Y Lin H Ma R Cai X. Role of Estrogen in Hepatocellular Carcinoma: Is Inflammation the Key? J Trans Med (2014) 12:93. doi: 10.1186/1479-5876-12-93 PMC3992128 24708807

[24] Ma WL Lai HC Yeh S Cai X Chang C. Androgen Receptor Roles in Hepatocellular Carcinoma, Fatty Liver, Cirrhosis and Hepatitis. Endocr Relat Cancer (2014) 21:R165–82. doi: 10.1530/ERC-13-0283 PMC4165608 24424503

[25] Yuan JM Ross RK Stanczyk FZ Govindarajan S Gao YT Henderson BE. A Cohort Study of Serum Testosterone and Hepatocellular Carcinoma in Shanghai, China. Int J Cancer (1995) 63:491–3. doi: 10.1002/ijc.2910630405 7591255

[26] Yu L Kubota H Imai K Yamaguchi M Nagasue N. Heterogeneity in Androgen Receptor Levels and Growth Response to Dihydrotestosterone in Sublines Derived From Human Hepatocellular Carcinoma Line (KYN-1). Liver (1997) 17:35–40. doi: 10.1111/j.1600-0676.1997.tb00776.x 9062878

[27] Wu MH Ma WL Hsu CL Chen YL Ou JH Ryan CK. Androgen Receptor Promotes Hepatitis B Virus-Induced Hepatocarcinogenesis Through Modulation of Hepatitis B Virus RNA Transcription. Sci Trans Med (2010) 2:32ra5. doi: 10.1126/scitranslmed.3001143 PMC3032595 20484730

[28] Ma WL Hsu CL Yeh CC Wu MH Huang CK Jeng LB. Hepatic Androgen Receptor Suppresses Hepatocellular Carcinoma Metastasis Through Modulation of Cell Migration and Anoikis. Hepatology (2012) 56:176–85. doi: 10.1002/hep.25644 PMC3673306 22318717

[29] Ma WL Jeng LB Lai HC Liao PY Chang C. Androgen Receptor Enhances Cell Adhesion and Decreases Cell Migration *via* Modulating Beta1-Integrin-AKT Signaling in Hepatocellular Carcinoma Cells. Cancer Lett (2014) 351:64–71. doi: 10.1016/j.canlet.2014.05.017 24944078

[30] Wang S Zheng W Ji A Zhang D Zhou M. Overexpressed miR-122-5p Promotes Cell Viability, Proliferation, Migration And Glycolysis Of Renal Cancer By Negatively Regulating Pkm2. Cancer Manag Res (2019) 11:9701–13. doi: 10.2147/CMAR.S225742 PMC6863119 31814765

[31] Ding FN Gao BH Wu X Gong CW Wang WQ Zhang SM. miR-122-5p Modulates the Radiosensitivity of Cervical Cancer Cells by Regulating Cell Division Cycle 25A (CDC25A). FEBS Open Bio (2019) 9(11):1869–79. doi: 10.1002/2211-5463.12730 PMC6823283 31505105

[32] Hernandez-Alcoceba R del Peso L Lacal JC. The Ras Family of GTPases in Cancer Cell Invasion. Cell Mol Life Sci (2000) 57(1):65–76. doi: 10.1007/s000180050499 10949581 PMC11146990

[33] Peng GL Tao YL Wu QN Zhang Y He JX. Positive Expression of Protein Chromosome 9 Open Reading Frame 86 (C9orf86) Correlated With Poor Prognosis in Non-Small Cell Lung Cancer Patients. J Thorac Dis (2016) 8(7):1449–59. doi: 10.21037/jtd.2016.04.70 PMC4958800 27499931

