# Supplementary figures and images for: Androgen Receptors Act as a Tumor Suppressor Gene to Suppress Hepatocellular Carcinoma Cells Progression via miR-122-5p/RABL6 Signaling
Source: Front Oncol. 2021 Oct 20;11:756779. doi: 10.3389/fonc.2021.756779 (PMC8564478; doi:10.3389/fonc.2021.756779)

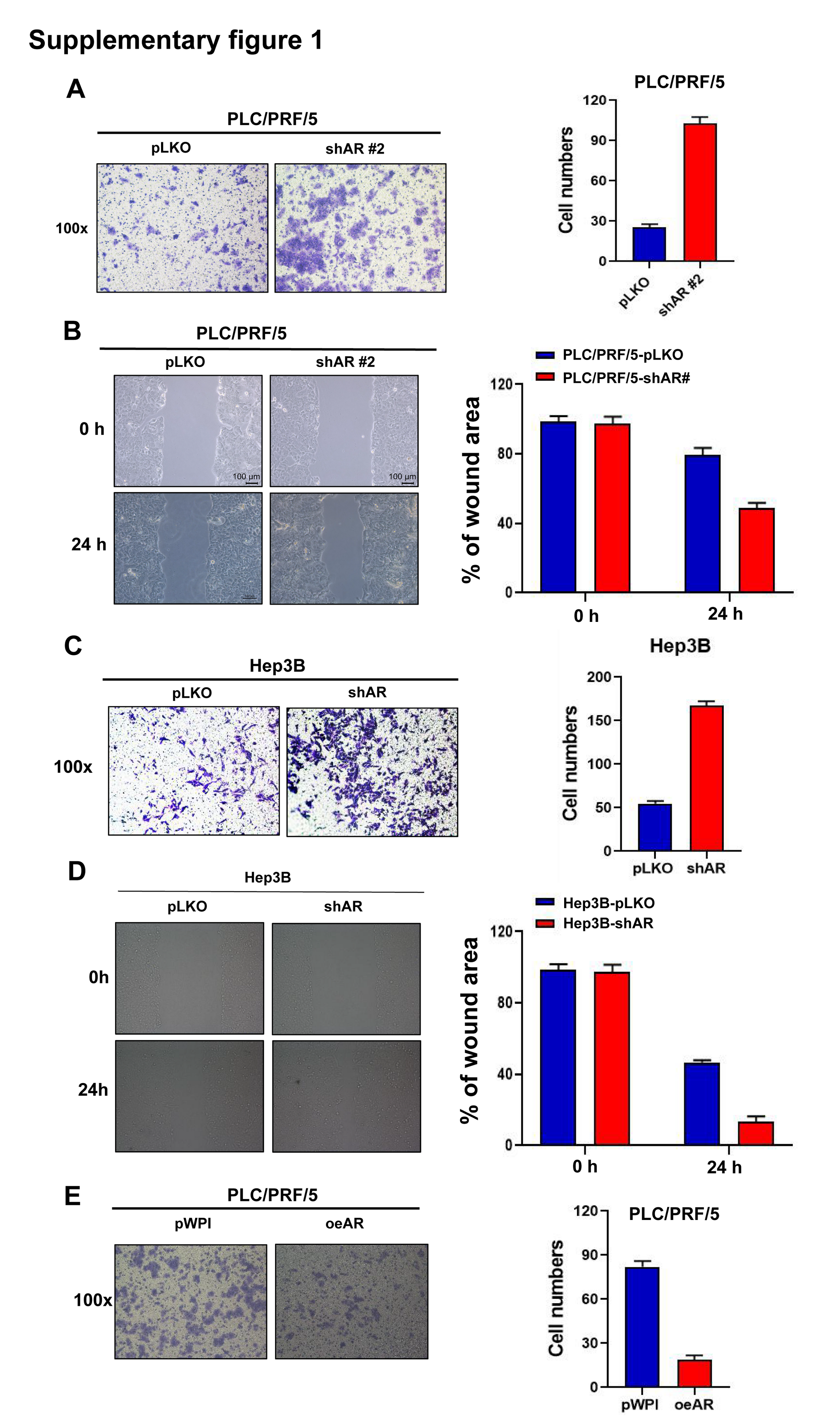

Supplement: Supplementary Figure 1 — (A) Transwell invasion assay was used to check the cells invasion capacity after transfecting the second shAR plasmid into the HCC PLC/PRF/5 cells. (B) Would healing migration assay was used to check the cells migration capacity after transfecting the second shAR plasmid into the HCC PLC/PRF/5 cells. (C) Transwell invasion assay was used to check the cells invasion capacity after knocking down AR in HCC Hep3B cells. (D) Would healing migration assay was used to check the cells migration capacity after knocking down AR in HCC Hep3B cells. (E) Transwell invasion assay was used to check the cells invasion capacity after overexpressing AR HCC PLC/PRF/5 cells. [file Image_1.tif]

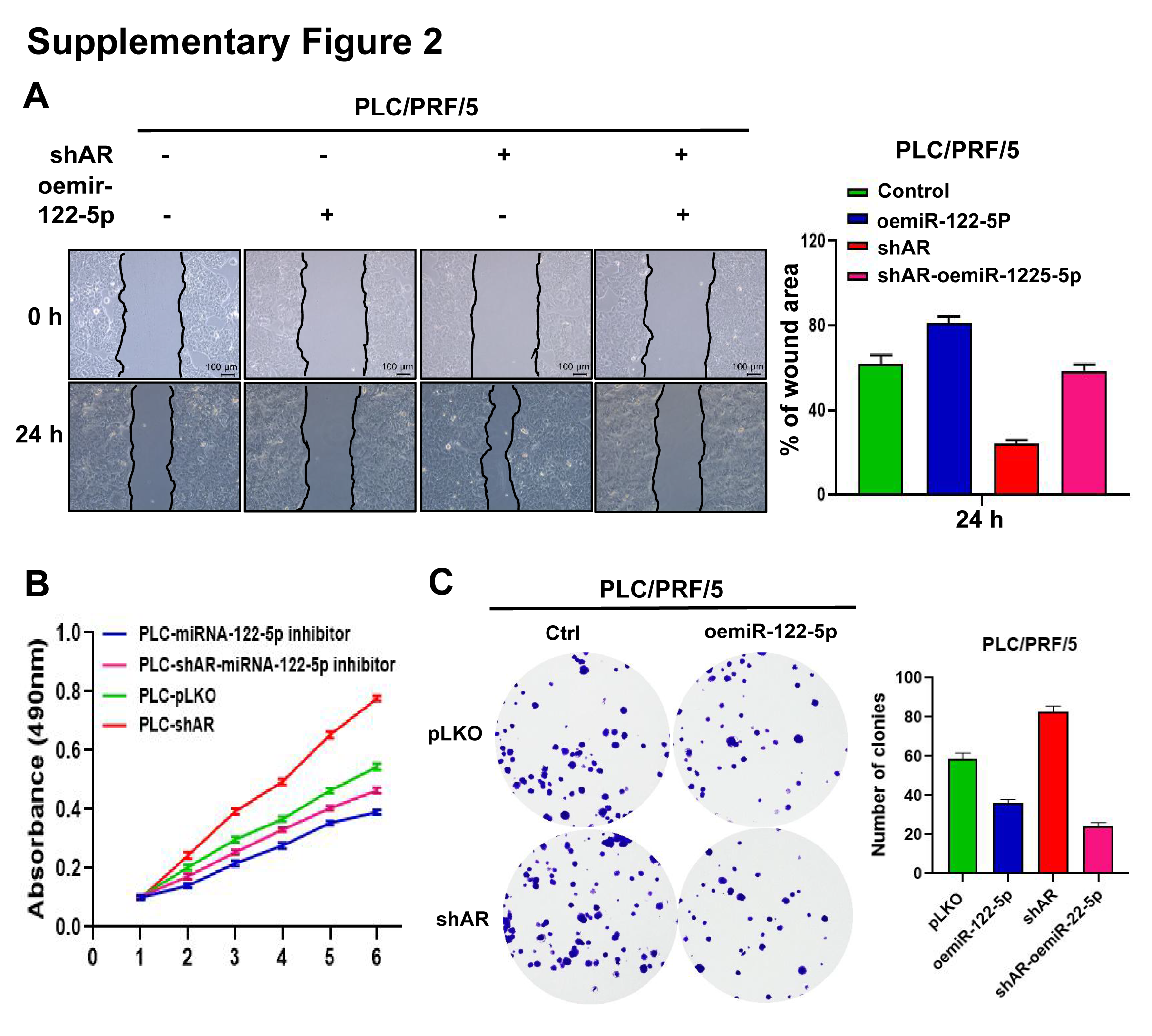

Supplement: Supplementary Figure 2 — (A) Wound healing migration assay was used to check HCC PLC/PRF/5 cells migration capacity after shAR/oemiR-122-5p in the cells. (B, C) MTT and colony formation assays were used to check HCC PLC/PRF/5 cells proliferation capacity after shAR/oemiR-122-5p in the cells. [file Image_2.tif]

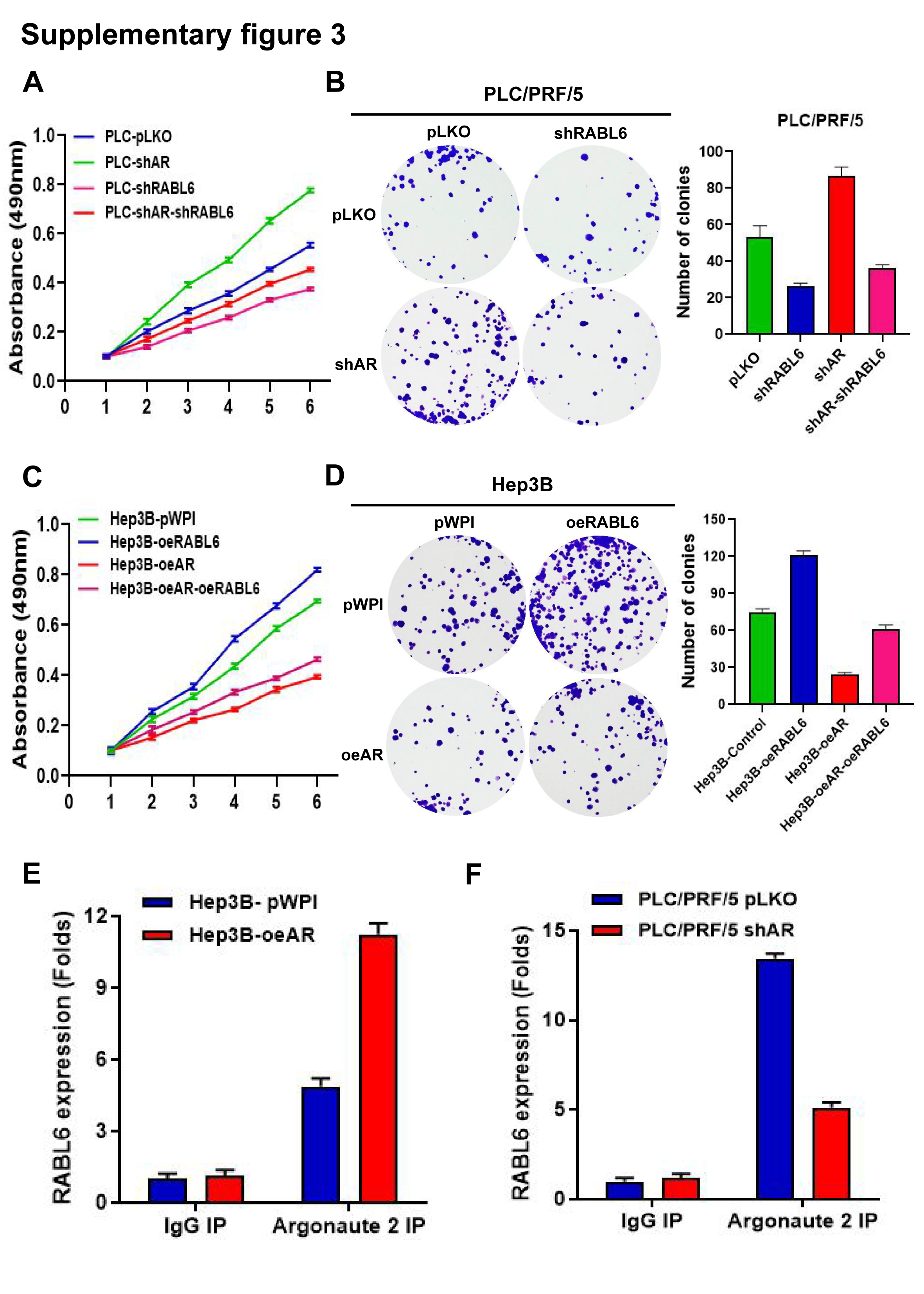

Supplement: Supplementary Figure 3 — (A, B) MTT and wound healing assays were used to check HCC PLC/PRF/5 cells proliferation capacity after shAR/shRABL6 in the cells. (C, D) MTT and wound healing assays were used to check HCC Hep3B cells invasion capacity after oeAR/oeRABL6 in the cells. (E, F) The RABL6 mRNA level was detected in Argonaute 2 complex using RNA interaction-precipitation (RIP) assay in Hep3B (E) cells transfected with pWPI or oeAR and in PLC/PRF/5 (F) cells transfected with pLKO or shAR. [file Image_3.tif]
